# Reproductive outcomes after expectant management and 5- versus 15-minute laparoscopic ethanol sclerotherapy for ovarian endometriomas: a retrospective cohort study

**DOI:** 10.3389/fendo.2026.1959548

**Published:** 2026-09-28

**Authors:** Yutong Wang, Qunxian Rao, Zhi Zeng, Manchao Li, Rang Liu, Rui Xiang, Feng Wang, Weifen Deng, Xiaoyan Liang, Jintao Peng, Haitao Zeng

**Affiliations:** 1 Reproductive Medicine Research Center, The Sixth Affiliated Hospital, Sun Yat-sen University, Guangzhou, China; 2 Guangdong Engineering Technology Research Center of Fertility Preservation, Guangzhou, Guangdong, China; 3 Biochemical Innovation Center, The Sixth Affiliated Hospital, Sun Yat-sen University, Guangzhou, Guangdong, China; 4 Department of Gynecologic Oncology, Sun Yat-sen Memorial Hospital, Sun Yat-sen University, Guangzhou, Guangdong, China; 5 Guangdong Provincial Clinical Research Center for Obstetrical and Gynecological Diseases, Guangzhou, Guangdong, China; 6 Fertility Center, Shenzhen Hengsheng Hospital, Shenzhen, Guangdong, China

**Keywords:** anti-müllerian hormone, cumulative live birth, ethanol sclerotherapy, inverse probability of treatment weighting, ovarian endometrioma, ovarian reserve

## Abstract

**Background:**

Laparoscopic ethanol sclerotherapy is a fertility-preserving alternative for ovarian endometriomas, but the appropriate ethanol exposure duration remains uncertain. We compared expectant management with 5- and 15-minute ethanol retention protocols.

**Methods:**

This retrospective cohort study included 333 infertile women with ovarian endometriomas ≥4 cm and complete cumulative reproductive follow-up: expectant management (n=125), 5-minute (n=124), or 15-minute (n=84) laparoscopic ethanol sclerotherapy. Multinomial propensity scores and inverse probability of treatment weighting (IPTW) balanced prespecified baseline covariates. The primary outcome was complete cumulative live birth; secondary outcomes included cumulative clinical pregnancy, embryological outcomes, and serum AMH changes. Additional sensitivity analyses examined FSH and adenomyosis, calendar year, male factor, and an analysis retaining all 357 clinically eligible women.

**Results:**

After primary weighting, all prespecified covariates were balanced (maximum absolute standardized mean difference, 0.094). Cumulative clinical pregnancy rates were 48.4%, 78.2%, and 58.1%, and cumulative live birth rates were 42.7%, 73.0%, and 50.8% in the expectant, 5-minute, and 15-minute groups, respectively (both overall P<0.001). Compared with expectant management, the 5-minute protocol was associated with higher odds of clinical pregnancy (OR = 3.84, 95% CI 1.86–7.93; P<0.001) and live birth (OR = 3.63, 95% CI 1.80–7.30; P<0.001), and with higher odds than the 15-minute protocol (OR = 2.60, 95% CI 1.13–6.00; P = 0.020; and OR = 2.62, 95% CI 1.18–5.81; P = 0.013). The 15-minute protocol did not differ significantly from expectant management. Doubly robust analyses were consistent. In the expanded sensitivity analyses and the additional sensitivity analysis retaining all 357 clinically eligible women, the direction of the primary outcome associations was also unchanged. The proportion of cycles yielding no transferable embryo was lower in the 5- and 15-minute groups than in the expectant group (10.7% and 9.4% vs. 32.8%; overall P<0.001). The relative AMH decline was greater after the 15-minute than the 5-minute protocol (−23.7% vs. −8.1%; P = 0.003).

**Conclusions:**

In this retrospective cohort, the 5-minute protocol was associated with higher cumulative pregnancy and live birth rates than expectant management and the 15-minute protocol. Both protocols were associated with fewer cycles yielding no transferable embryo than expectant management, whereas 15-minute retention was associated with a greater AMH decline. These findings are hypothesis-generating and require prospective randomized confirmation.

## Introduction

Endometriosis is a chronic estrogen-dependent inflammatory disease affecting approximately 5–10% of women of reproductive age and is an important cause of pain and infertility (1). Ovarian endometriomas (OEMs), which occur in 17–44% of patients with endometriosis, have been associated with reduced ovarian reserve, impaired oocyte and embryo quality, and altered endometrial receptivity (2–4).

For women seeking pregnancy, laparoscopic cystectomy may be considered in selected cases but can reduce ovarian reserve through inadvertent removal of healthy ovarian tissue (5). Substantial postoperative reductions in serum anti-Müllerian hormone (AMH) have been reported after endometrioma surgery (6). Ethanol sclerotherapy has therefore emerged as a fertility-preserving alternative that induces aseptic inflammation and fibrosis of the cyst lining (7). Previous studies have reported favorable therapeutic and ovarian-reserve outcomes after ethanol sclerotherapy, although the procedures and exposure protocols have varied (8–10). Potential complications include ethanol leakage, bleeding, chemical peritonitis, and injury to adjacent structures (11). Compared with transvaginal or percutaneous approaches, laparoscopic sclerotherapy permits direct visualization, controlled ethanol instillation and aspiration, and assessment of coexisting pelvic disease (12, 13). Nevertheless, current ESHRE guidelines do not recommend routine surgery for endometriomas before assisted reproductive technology (ART), primarily because of concerns regarding treatment-related ovarian damage (14).

The optimal duration of ethanol retention remains uncertain. Reported exposure times range from 1 to 30 minutes, with 10–15 minutes commonly used, although shorter protocols may achieve comparable outcomes (15–18). A related question is whether an endometrioma should be treated before ovarian stimulation. Expectant management avoids iatrogenic ovarian damage, whereas an untreated endometrioma may adversely affect the follicular environment and fertilization capacity (3, 14, 19).

The reproductive effect of sclerotherapy may therefore depend on the balance between treatment of the endometrioma and ethanol-related ovarian injury, which may in turn be influenced by exposure duration. However, expectant management and laparoscopic sclerotherapy using different ethanol retention times have not been compared simultaneously.

## Materials and methods

### Ethics

This study was approved by the Ethics Committee of the Sixth Affiliated Hospital, Sun Yat-sen University (approval no. 2024ZSLYEC-604), and the procedures were performed in accordance with the ethical standards of the Declaration of Helsinki. The requirement for informed consent was waived because of the retrospective nature of the study.

### Study cohorts

This retrospective cohort study was conducted at the Reproductive Medicine Research Center, the Sixth Affiliated Hospital, Sun Yat-sen University, from June 2019 to April 2024. Women with infertility and ultrasonographically diagnosed unilateral or bilateral ovarian endometriomas were screened for eligibility.

Ovarian endometrioma was diagnosed according to the ESHRE guideline on the basis of ground-glass echogenicity, one to four compartments, and no papillary structures with detectable blood flow (20). Ultrasound measurements were obtained by qualified ultrasonographers as part of the pretreatment clinical assessment. In the surgical groups, this assessment was completed within 1 week before surgery. Clinical reports documented three orthogonal cyst diameters. Endometrioma cyst size was defined as the longest diameter; for bilateral or multiple endometriomas, the largest recorded diameter was used. Adenomyosis was diagnosed by transvaginal ultrasonography according to the revised Morphological Uterus Sonographic Assessment (MUSA) consensus features of adenomyosis (21).

Inclusion required an ovarian endometrioma cyst size ≥4 cm and subsequent controlled ovarian stimulation. Complete reproductive follow-up was required for the primary cumulative-outcome analysis. Exclusion criteria were age >45 years, polycystic ovary syndrome, a known chromosomal abnormality in either partner, uterine factors (congenital anomalies, submucosal myoma, endometrial hyperplasia or polyps, or intrauterine adhesions), previous ovarian surgery, recurrent spontaneous abortion or recurrent implantation failure. Follow-up was considered complete when the woman had a live birth or when all embryos derived from the index retrieval had been used. Among 357 clinically eligible women, 24 had incomplete cumulative follow-up because unused cryopreserved embryos remained at database closure; these women were excluded from the primary complete-outcome analysis, leaving 333 women: 125 with expectant treatment, 124 with a 5-minute sclerotherapy, and 84 with a 15-minute sclerotherapy.

Patients underwent expectant management without surgery, 5-minute or 15-minute laparoscopic ethanol sclerotherapy before ovarian stimulation. Management was selected through individualized clinician-patient shared decision-making rather than a prespecified allocation algorithm. The available records captured clinical information that could be relevant to treatment choice, including cyst size and laterality, patient age, ovarian reserve and other hormonal measures, and infertility characteristics. Clinical judgment and patient preference could also influence the choice, but the specific decision process, discussion content, and timing were not recorded in the electronic medical record.

### Surgical procedures

The 5-minute and 15-minute protocols were used contemporaneously during the study period and did not represent a planned sequential replacement. All sclerotherapy procedures were performed by the same experienced surgical team using an otherwise standardized technique.

In the sclerotherapy groups, baseline serum AMH, follicle-stimulating hormone (FSH), and luteinizing hormone (LH) were measured on menstrual cycle days 2–3, and surgery was performed 3–7 days after menstruation. Corresponding baseline hormones were measured on cycle days 2–3 in the expectant group.

Laparoscopic sclerotherapy was performed under general anesthesia using a standard three-port approach based on previously reported procedures (12, 22). A 10-mm umbilical port and two 5-mm lateral ports were placed. After pelvic inspection, a small incision was made in the endometrioma using a circular monopolar coagulator. The cyst contents were aspirated, and the cavity was repeatedly irrigated using a 14-gauge laparoscopic puncture needle connected to an irrigation–suction system (HiQ+; Olympus Winter & Ibe GmbH, Hamburg, Germany). The cyst volume was estimated from the aspirated fluid, and dehydrated ethanol (China National Medicines Guorui Pharmaceutical Co., Ltd., China) equivalent to approximately 80% of this volume was instilled and retained for 5 or 15 minutes. The ethanol was then completely aspirated, and the cavity was thoroughly rinsed with sterile saline. A cyst-wall biopsy was obtained and submitted for histopathological examination. The internal cyst wall was inspected for features suggestive of neoplastic change. No cystectomy or ablation was performed. Hemostasis was confirmed before standard port closure. Disease severity was staged intraoperatively according to the revised American Society for Reproductive Medicine (rASRM) classification and is reported descriptively for the surgical groups.

### ART procedures

Controlled ovarian hyperstimulation (COH) was initiated approximately 2 months after sclerotherapy in the two sclerotherapy groups. In the expectant group, COH was initiated according to the routine clinical schedule after enrolment. At the initiation of ART, postoperative serum AMH was measured in the two sclerotherapy groups. Preoperative and postoperative AMH concentrations were measured in the hospital central laboratory using the cobas e 601 platform and reported as official laboratory results.

All patients received a single intramuscular injection of a GnRH agonist (Diphereline; Ipsen, France; 3.75 mg) on menstrual cycle days 2–4 for pituitary downregulation. After 28–42 days, ovarian stimulation was initiated with recombinant FSH (r-FSH; Lizhu, China) or human menopausal gonadotropin (HMG; Lizhu, China) if serum FSH and LH concentrations were both <5 IU/L. The initial gonadotropin dose was 150–225 IU/day and was individualized according to age, body mass index, and ovarian reserve. The dose was subsequently adjusted according to follicular development on transvaginal ultrasound and serial serum hormone measurements. When one follicle reached 18 mm or two follicles reached 17 mm, ovulation was triggered with 6,000–10,000 IU human chorionic gonadotropin (hCG; Lizhu, China). Oocyte retrieval was performed 36–38 hours later. IVF or ICSI was selected according to semen parameters and previous fertilization outcomes.

Fresh or frozen-thawed cleavage-stage embryos or blastocysts were transferred based on clinical and embryological assessment. The decision to perform a fresh or frozen embryo transfer (FET) was made at the discretion of the treating physician. Fresh transfer was cancelled and all embryos were cryopreserved in cases of risk of ovarian hyperstimulation syndrome, hydrosalpinx, or endometrial asynchrony.

In fresh transfer cycles, cleavage-stage embryos were transferred on day 3 after oocyte retrieval, and blastocysts on day 5. Luteal phase support (LPS) was initiated on the day of oocyte retrieval and comprised intramuscular progesterone (Zhejiang Xianju, China, 40mg/d) combined with vaginal progesterone (Utrogestan, Besins, France, 200mg/d).

For FET, endometrial preparation was individualized according to each patient’s clinical characteristics, including menstrual regularity, ovarian function, and underlying uterine conditions. Four protocols were employed: natural cycle (NC), mild ovarian stimulation cycle, hormone replacement therapy (HRT), and GnRH agonist combined with HRT (GnRH-a+HRT). In NC and mild stimulation cycles, embryo transfer was performed 3 days after confirmed ovulation for cleavage-stage embryos, or 5 days for blastocysts. In HRT cycles, estradiol valerate (Progynova, Bayer, Germany, 6–8mg/d) was initiated from cycle day 3, and dydrogesterone (Dapotone, Abbott, The Netherlands, 20 mg twice daily) was added once endometrial thickness exceeded 8 mm; cleavage-stage embryos were transferred 3 days after the initiation of progesterone supplementation, and blastocysts on day 5. In GnRH-a+HRT cycles, triptorelin 3.75 mg (Diphereline; Ipsen, France) was administered on cycle day 2–3; HRT was initiated approximately 28 days later when pituitary suppression was confirmed (FSH and LH each <5 IU/L and endometrial thickness <5 mm), followed by the same HRT protocol as described above.

For all FET cycles, LPS comprised intramuscular progesterone (Zhejiang Xianju, China, 40mg/d) combined with vaginal progesterone (Utrogestan, Besins, France, 200mg/d), initiated on the day of embryo transfer. If pregnancy was confirmed, LPS was continued until approximately 9–12 weeks of gestation in both fresh and frozen transfer cycles, with the exact duration adjusted according to individual clinical circumstances. In HRT and GnRH-a+HRT cycles, estradiol valerate was also maintained until approximately 10–12 weeks of gestation, given the absence of an endogenous corpus luteum.

### Outcome measures

The primary outcome was the complete cumulative live birth rate per woman after one initiated ovarian stimulation cycle (index oocyte retrieval), defined as the proportion of women who achieved at least one live birth from all fresh and frozen-thawed embryo transfers derived from that retrieval. Follow-up duration was calculated from the date of index oocyte retrieval to the earliest of first live birth, use of all embryos derived from the index retrieval, or database closure (December 31, 2025). Women who retained unused cryopreserved embryos without a live birth at database closure were considered to have incomplete cumulative follow-up and were excluded from the primary complete-outcome analysis.

Secondary outcomes included the cumulative clinical pregnancy rate, oocyte yield, embryological outcomes, transferable embryo availability, and AMH changes after sclerotherapy. Cumulative clinical pregnancy was defined as at least one clinical pregnancy resulting from all embryo transfers derived from the index retrieval. Clinical pregnancy was defined as the ultrasonographic visualization of at least one intrauterine gestational sac with fetal cardiac activity. Live birth was defined as delivery of at least one infant showing signs of life at or after 24 completed weeks of gestation.

Normal fertilization was identified by the presence of two pronuclei (2PN). In our routine clinical practice, oocyte maturity was not assessed before conventional IVF insemination, whereas mature oocytes were identified before injection in ICSI cycles. Therefore, 2PN yield per retrieved oocyte was calculated as the number of 2PN zygotes divided by the number of oocytes retrieved. The 2PN cleavage rate was calculated as the number of cleaved embryos derived from 2PN zygotes divided by the number of 2PN zygotes. A day-3 embryo was considered transferable if it had more than four cells with <20% fragmentation or four cells without fragmentation; embryos with six to nine cells and <20% fragmentation were classified as high quality. The transferable and high-quality day-3 embryo rates were calculated using the number of cleaved embryos derived from 2PN zygotes as the denominator.

Embryos selected for continued culture rather than transfer or cryopreservation on day 3 constituted the extended-culture denominator. Blastocysts were graded using the Gardner system. Blastocysts graded 3CC or better were considered transferable, and those graded 3BB or better were considered high quality. The transferable blastocyst formation rate was calculated as the number of transferable blastocysts divided by the number of embryos entering extended culture, whereas the high-quality blastocyst rate was calculated as the number of high-quality blastocysts divided by the number of transferable blastocysts. A rate was treated as missing when its denominator was zero.

The total transferable embryo count comprised day-3 embryos retained for transfer or cryopreservation and transferable blastocysts obtained after extended culture, with each embryo counted once according to its final developmental disposition. A cycle yielding no transferable embryo was defined as one in which no embryo was ultimately available for transfer or cryopreservation. Absolute AMH change was calculated as postoperative minus preoperative AMH, and relative AMH change as (postoperative AMH − preoperative AMH)/preoperative AMH × 100%. AMH change was assessed only in the two sclerotherapy groups because a corresponding follow-up measurement was unavailable in the expectant group.

### Statistical analysis

Continuous and categorical variables are presented as mean ± standard deviation and n (%), respectively. Baseline continuous variables were compared using one-way analysis of variance (ANOVA) when the homogeneity-of-variance assumption was satisfied and Welch’s ANOVA when it was not. Homogeneity of variance was assessed using Levene’s test. Categorical variables were compared using chi-square tests.

Generalized propensity scores were estimated using multinomial logistic regression including age, body mass index (BMI), infertility duration, AMH level, endometrioma cyst size, cyst laterality, and infertility type. Stabilized inverse probability of treatment weights targeting the average treatment effect were calculated as the marginal probability of the treatment received divided by the corresponding individual propensity score. Weights were truncated at the 1st and 99th percentiles. Covariate balance was assessed using the maximum absolute standardized mean difference (SMD) across the three pairwise group comparisons, with values <0.1 considered to indicate adequate balance. Effective sample sizes were also calculated after weighting. Baseline FSH and adenomyosis were jointly included in an expanded sensitivity propensity-score model in the 333 women with complete cumulative outcomes. Calendar year, defined as the year recorded for the index oocyte retrieval, was evaluated as a categorical covariate in a separate sensitivity propensity-score model. Male factor was included as an additional binary sensitivity covariate in a third model in the same 333 women. The conservative lower-bound analysis in all 357 clinically eligible women used the original primary propensity-score covariates. rASRM stage was available only for the surgical groups and was reported descriptively. Male factor, stimulation characteristics, embryo-transfer characteristics, and follow-up information were otherwise reported descriptively and were not included in the primary propensity-score model. Standardized pre-treatment AFC measurements and complete previous ART records were unavailable.

Outcomes were analyzed using survey-weighted generalized linear models. Embryological counts and patient-level embryological rates were analyzed using Gaussian models with an identity link. The number of oocytes retrieved was analyzed using inverse probability of treatment weighting (IPTW) alone because it preceded fertilization, whereas counts from the 2PN stage onward and all embryological rates were additionally adjusted for fertilization method (IVF or ICSI). Patient-level rates with a zero denominator were treated as missing. The proportion of cycles yielding no transferable embryo was analyzed using an IPTW-weighted quasibinomial model with a logit link, without additional adjustment for fertilization method.

Complete cumulative clinical pregnancy and complete cumulative live birth were analyzed using IPTW-weighted quasibinomial models with a logit link. Overall group differences were evaluated using design-based Wald tests. Tukey-adjusted pairwise comparisons based on estimated marginal means were performed for complete cumulative clinical pregnancy, complete cumulative live birth, and the absence of transferable embryos. Odds ratios (ORs) and 95% confidence intervals (CIs) were reported for pairwise comparisons. Separate sensitivity models additionally adjusted the propensity score and corresponding doubly robust outcome model for FSH and adenomyosis, categorical calendar year, or male factor, respectively. A conservative lower-bound sensitivity analysis retained all 357 clinically eligible women. All outcomes from transfer cycles completed before database closure were observed. For women without a live birth who retained unused cryopreserved embryos at database closure, the potential contribution of future transfers to cumulative live birth was conservatively assumed to be zero. This analysis provides a worst-case lower bound for the cumulative live-birth outcome and does not eliminate informative follow-up. Fresh and frozen-thawed transfer-level outcomes were also summarized descriptively in the same 333 women.

Doubly robust analyses of complete cumulative clinical pregnancy and live birth combined IPTW with additional outcome-model adjustment for age, BMI, infertility duration, baseline AMH, endometrioma cyst size, cyst laterality, and infertility type. The sensitivity outcome models additionally included baseline FSH and adenomyosis, categorical calendar year, or male factor, respectively. Between-group comparisons of preoperative AMH, postoperative AMH, and absolute and relative AMH changes were performed using Student’s t-test or Welch’s t-test according to the result of Levene’s test. Within-group preoperative-to-postoperative AMH changes were evaluated using paired t-tests. All tests were two-sided, and P<0.05 was considered statistically significant. Analyses were performed using R version 4.4.2.

## Results

### Baseline characteristics and covariate balance

A total of 357 women were clinically eligible: 136 in the expectant group, 133 in the 5-minute group, and 88 in the 15-minute group. 24 women had incomplete cumulative follow-up because unused cryopreserved embryos remained at database closure. The primary complete-outcome analysis therefore included 333 women: 125, 124, and 84 in the three groups, respectively (details in **Figure 1**). All ovarian endometriomas were diagnosed by ultrasonography, and cyst-wall specimens from the sclerotherapy groups underwent histopathological examination. No severe procedure-related complications occurred. Before weighting, age, infertility type, endometrioma cyst size, and cyst laterality differed among groups; after IPTW, all 7 covariates in the primary model were balanced (**Table 1**). Effective sample sizes were 101, 118, and 58, respectively. rASRM stage III or IV disease predominated in both surgical groups (**Supplementary Table S2**).

**Figure 1:**
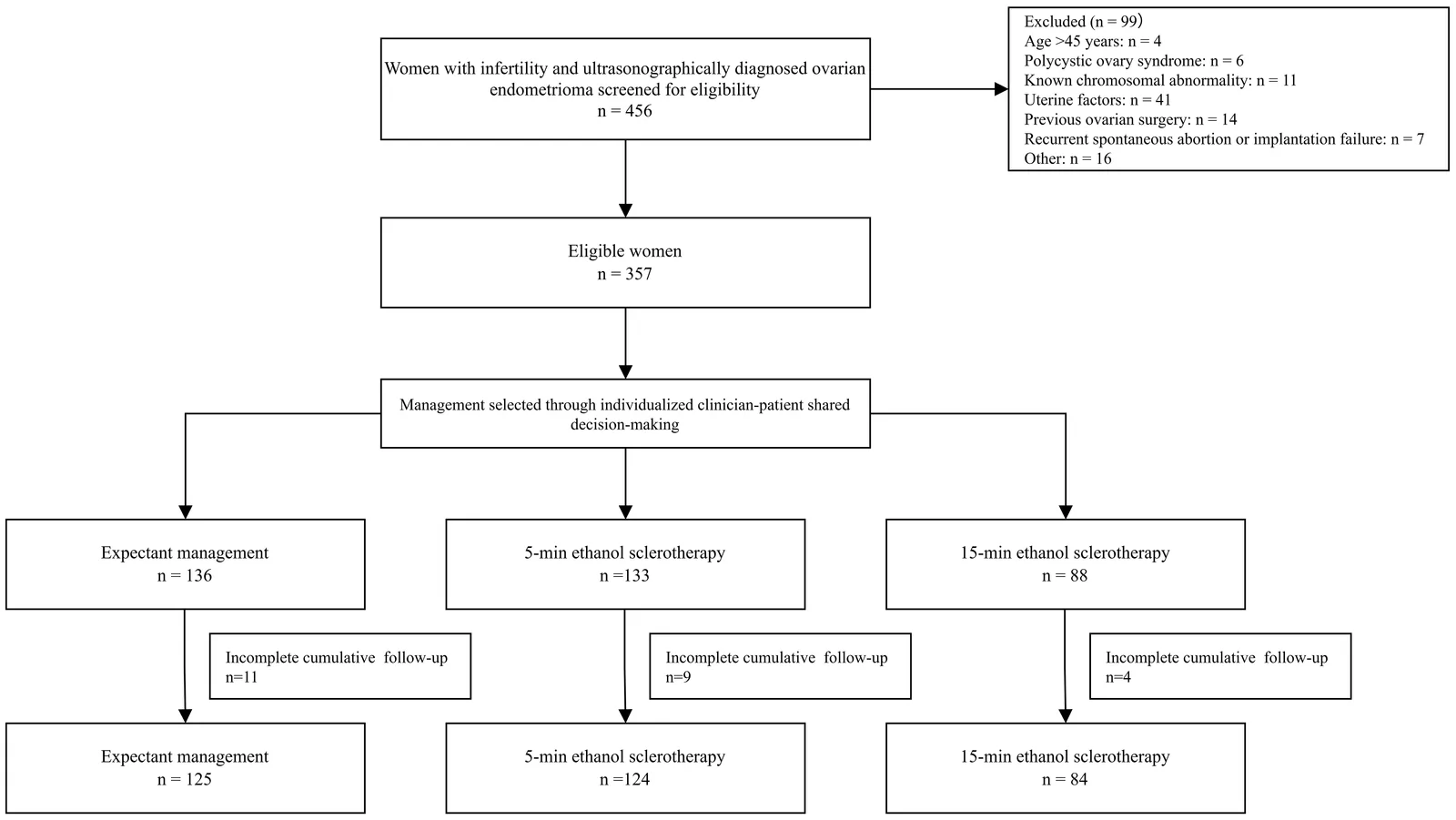
Flowchart of participant screening, management selection, and the complete cumulative-outcome cohort. Of 357 clinically eligible women, 24 had incomplete cumulative follow-up because unused cryopreserved embryos remained at database closure, leaving 333 women for the primary analysis.

**Table 1:** Baseline characteristics and covariate balance before and after IPTW.

Characteristic	Expectant (n=125)	5-min (n=124)	15-min (n=84)	P value*	Max SMD before	Max SMD after
Age (years)	34.30 ± 4.00	32.35 ± 3.95	31.95 ± 4.04	<0.001	0.589	0.035
BMI (kg/m²)	21.34 ± 2.89	21.58 ± 2.86	21.08 ± 2.43	0.451	0.180	0.076
AMH (ng/mL)	1.86 ± 1.73	2.11 ± 1.28	1.96 ± 1.33	0.428	0.167	0.094
Basal FSH (IU/L)	8.14 ± 2.73	7.76 ± 2.82	7.83 ± 2.08	0.311	—	—
Infertility duration (years)	3.88 ± 2.93	3.73 ± 3.05	3.61 ± 2.54	0.791	0.096	0.093
Infertility type, n (%)				0.027	0.172	0.057
Primary infertility	70 (56.0)	70 (56.5)	33 (39.3)			
Secondary infertility	55 (44.0)	54 (43.5)	51 (60.7)			
Endometrioma cyst size	5.33 ± 1.04	5.53 ± 1.42	5.80 ± 1.58	0.049†	0.345	0.074
Cyst laterality				<0.001	0.272	0.017
Unilateral	98 (78.4)	89 (71.8)	43 (51.2)			
Bilateral	27 (21.6)	35 (28.2)	41 (48.8)			
Adenomyosis, n (%)	25 (20.0)	23 (18.5)	15 (17.9)	0.919	—	—
Male factor, n (%)	13 (10.4)	12 (9.7)	7 (8.3)	0.883	—	—

Data are mean ± SD or n (%). P values were calculated from the unweighted data and are descriptive. *One-way ANOVA (chi-square for categorical variables) on unweighted data. ^†^Welch’s ANOVA (Levene's P<0.05). SMD, standardized mean difference (maximum across the three pairwise comparisons); absolute SMDs <0.1 after IPTW indicate adequate balance for variables included in the primary IPTW model. FSH, follicle-stimulating hormone; AMH, anti-Müllerian hormone; BMI, body mass index; IPTW, inverse probability of treatment weighting. For FSH, adenomyosis, and male factor, dashes in the SMD columns indicate that these variables were not included in the primary IPTW model; these variables were evaluated in the expanded sensitivity analysis, whereas male factor was evaluated in an additional sensitivity analysis.

### ART-cycle characteristics

Total gonadotropin dose and stimulation duration differed among groups (P = 0.007 and P<0.001, respectively), whereas IVF/ICSI distribution did not (P = 0.347). Fresh/frozen-thawed, cleavage-stage/blastocyst, and single/double embryo-transfer characteristics are reported in **Supplementary Table S1**, Panel A. In the exploratory transfer-level analysis of the 333 women with complete cumulative outcomes, there were 148 fresh and 218 frozen-thawed transfer cycles. Pathway-specific clinical pregnancy and live-birth outcomes, with their respective transfer-cycle denominators, are reported descriptively in **Supplementary Table S1**, Panel B.

### Embryological outcomes

The number of oocytes retrieved did not differ significantly among the three groups (overall P = 0.325; **Table 2**). Significant overall differences were observed in the numbers of 2PN zygotes and cleaved embryos, as well as in the 2PN yield per retrieved oocyte, 2PN cleavage, and high-quality day-3 embryo rates. The weighted high-quality day-3 embryo rates were 65.7%, 51.4%, and 63.7% in the expectant, 5-minute, and 15-minute groups, respectively (overall P = 0.009). No significant overall differences were observed in the other embryological counts or rates, including the total number of transferable embryos.

**Table 2:** IPTW-weighted embryological outcomes.

Outcome	Expectant	5-min	15-min	Overall P*
Embryological counts				
No. of oocytes retrieved	7.81 ± 0.75	8.94 ± 0.61	7.61 ± 0.79	0.325
No. of 2PN zygotes	4.01 ± 0.52	6.15 ± 0.43	5.87 ± 0.69	0.004
No. of cleaved embryos derived from 2PN zygotes	3.93 ± 0.52	5.72 ± 0.41	5.33 ± 0.59	0.020
No. of transferable day-3 embryos	3.39 ± 0.49	4.76 ± 0.36	4.42 ± 0.50	0.068
No. of high-quality day-3 embryos	2.84 ± 0.45	3.17 ± 0.28	3.64 ± 0.46	0.560
No. of transferable blastocysts	1.79 ± 0.35	2.18 ± 0.24	2.04 ± 0.35	0.622
No. of high-quality blastocysts	1.18 ± 0.27	1.42 ± 0.18	1.21 ± 0.26	0.642
Embryological ratios				
2PN yield per retrieved oocyte (%)	49.3 ± 3.7	72.6 ± 2.6	77.7 ± 3.2	<0.001
2PN cleavage rate (%)	97.5 ± 0.9	92.8 ± 1.6	93.7 ± 1.7	0.016
Transferable day-3 embryo rate (%)	85.3 ± 2.4	81.3 ± 2.6	84.5 ± 2.8	0.604
High-quality day-3 embryo rate (%)	65.7 ± 3.9	51.4 ± 3.0	63.7 ± 4.4	0.009
Transferable blastocyst formation rate (%)	75.7 ± 3.8	66.4 ± 3.0	66.9 ± 5.5	0.112
High-quality blastocyst rate (%)	57.0 ± 5.9	64.7 ± 3.6	57.1 ± 7.2	0.344
Embryo availability				
No. of transferable embryos	2.86 ± 0.39	3.76 ± 0.27	3.37 ± 0.41	0.139
No transferable embryo (%)	32.8 ± 4.5	10.7 ± 3.1	9.4 ± 2.8	<0.001

Data are presented as IPTW-weighted descriptive means or proportions ± standard errors. *Overall P values were obtained using Wald tests from IPTW-weighted generalized linear models. The number of oocytes retrieved and the proportion of cycles yielding no transferable embryo were analyzed using IPTW alone; embryological outcomes from the 2PN stage onward were additionally adjusted for fertilization method (IVF or ICSI). 2PN yield per retrieved oocyte was calculated as the number of 2PN zygotes divided by the number of retrieved oocytes; it is not a conventional fertilization rate based on inseminated or injected mature oocytes. The transferable and high-quality day-3 embryo rates were calculated using the number of cleaved embryos derived from 2PN zygotes as the denominator. The transferable blastocyst formation rate was calculated using the number of embryos entering extended culture as the denominator, and the high-quality blastocyst rate using the number of transferable blastocysts as the denominator. Blastocysts graded 3CC or better and 3BB or better were considered transferable and high quality, respectively. In Tukey-adjusted comparisons, the expectant group had higher odds of yielding no transferable embryo than the 5-minute group (OR = 4.08, 95% CI 1.66–9.99; P<0.001) and the 15-minute group (OR = 4.69, 95% CI 1.90–11.59; P<0.001), with no significant difference between the sclerotherapy groups (OR = 1.15, 95% CI 0.39–3.37; P = 0.950). 2PN, two pronuclei; IPTW, inverse probability of treatment weighting; OR, odds ratio; CI, confidence interval; IVF, *in vitro* fertilization; ICSI, intracytoplasmic sperm injection.

The proportion of cycles yielding no transferable embryo was lower in both sclerotherapy groups than in the expectant group (10.7% and 9.4% vs. 32.8%; overall P<0.001). In Tukey-adjusted comparisons, the expectant group had higher odds of yielding no transferable embryo than the 5-minute group (OR = 4.08, 95% CI 1.66–9.99; P<0.001) and the 15-minute group (OR = 4.69, 95% CI 1.90–11.59; P<0.001). No significant difference was observed between the two sclerotherapy groups (OR = 1.15, 95% CI 0.39–3.37; P = 0.950).

### Primary reproductive outcomes

Cumulative clinical pregnancy rates were 48.4%, 78.2%, and 58.1% in the expectant, 5-minute, and 15-minute groups, respectively, and cumulative live birth rates were 42.7%, 73.0%, and 50.8% (both overall P<0.001; **Table 3**). The 5-minute protocol was associated with higher odds of clinical pregnancy than expectant management (OR = 3.84, 95% CI 1.86–7.93; P<0.001) and the 15-minute protocol (OR = 2.60, 95% CI 1.13–6.00; P = 0.020). Corresponding ORs for live birth were 3.63 (95% CI 1.80–7.30; P<0.001) and 2.62 (95% CI 1.18–5.81; P = 0.013). The 15-minute protocol did not differ significantly from expectant management for either outcome.

**Table 3:** IPTW-weighted primary reproductive outcomes and doubly robust analysis.

A. Weighted rates and pairwise comparisons							
Outcome	Expectant	5-min	15-min	Overall P	Comparison	OR (95% CI)	P value*
Cumulative clinical pregnancy (%)	48.4	78.2	58.1	<0.001	5-min vs. Expectant	3.84 (1.86–7.93)	<0.001
					5-min vs. 15-min	2.60 (1.13–6.00)	0.020
					15-min vs. Expectant	1.48 (0.68–3.22)	0.472
Cumulative live birth (%)	42.7	73.0	50.8	<0.001	5-min vs. Expectant	3.63 (1.80–7.30)	<0.001
					5-min vs. 15-min	2.62 (1.18–5.81)	0.013
					15-min vs. Expectant	1.39 (0.64–3.02)	0.585
B. Doubly robust models (reference = expectant)							
Variable	CPR β (SE)		P value		CLBR β (SE)	P value	
5-min group	1.559 (0.339)		<0.001		1.544 (0.331)	<0.001	
15-min group	0.406 (0.356)		0.255		0.372 (0.360)	0.302	
Age	−0.061 (0.044)		0.159		−0.068 (0.041)	0.099	
BMI	−0.001 (0.054)		0.992		0.007 (0.053)	0.893	
Infertility duration	−0.074 (0.052)		0.150		−0.071 (0.049)	0.150	
Baseline AMH	0.445 (0.120)		<0.001		0.510 (0.114)	<0.001	
Endometrioma cyst size	−0.185 (0.105)		0.078		−0.169 (0.104)	0.107	
Cyst laterality	0.056 (0.315)		0.859		0.124 (0.324)	0.702	
Infertility type	−0.207 (0.283)		0.464		−0.086 (0.283)	0.762	

*Pairwise comparisons were obtained from estimated marginal means, with Tukey adjustment for multiple comparisons. Pairwise ORs are presented in the direction shown; an OR >1 favors the first-listed group. Doubly robust models were IPTW-weighted quasibinomial generalized linear models additionally adjusted for age, BMI, infertility duration, AMH, endometrioma cyst size cyst laterality, and infertility type. Regression coefficients are presented on the log-odds scale, with expectant management as the reference group. CPR, cumulative clinical pregnancy rate; CLBR, cumulative live birth rate; OR, odds ratio; CI, confidence interval; AMH, anti-Müllerian hormone; IPTW, inverse probability of treatment weighting.

Doubly robust analyses yielded consistent findings: the 5-minute protocol remained positively associated with clinical pregnancy and live birth relative to expectant management, whereas the 15-minute protocol did not (**Table 3**). Expanded baseline-covariate, calendar-year, and male-factor sensitivity analyses all retained the direction and approximate magnitude of the primary outcome associations. Complete sensitivity estimates are presented in **Supplementary Table S3**, and model-level balance, weight, ESS, and overlap diagnostics are presented together in **Supplementary Table S4**.

Among the 357 clinically eligible women, median follow-up duration was 123 days (IQR, 12–346) in the expectant group, 332 days (IQR, 302–363) in the 5-minute group, and 303 days (IQR, 94.8–342.2) in the 15-minute group. In the expectant group, 47/136 women (34.6%) had no embryo transfer; their follow-up was 7–14 days (median, 10 days), and none had a clinical pregnancy. Among women with any embryo transfer, follow-up was shorter in those without a clinical pregnancy than in those with a clinical pregnancy (median, 113 vs. 346 days), indicating potentially outcome-dependent ascertainment of cumulative outcomes (**Supplementary Table S6**). In the conservative lower-bound analysis retaining all 357 women, weighted cumulative clinical pregnancy rates were 50.2%, 78.6%, and 59.2%, and weighted cumulative live birth rates were 37.2%, 68.3%, and 49.2% in the expectant, 5-minute, and 15-minute groups, respectively. The direction of the primary associations was unchanged. This analysis used a conservative worst-case lower-bound assumption that future transfers from unused cryopreserved embryos would make no additional contribution to cumulative live birth and does not eliminate informative follow-up (**Supplementary Table S3**).

### Change in ovarian reserve (AMH)

Preoperative AMH was comparable between the sclerotherapy groups (P = 0.440; **Table 4**) and declined significantly after both protocols. The median interval from surgery to postoperative AMH assessment was 59.0 days (IQR, 52.0–66.25) in the 5-minute group and 54.5 days (IQR, 49.0–64.0) in the 15-minute group (**Supplementary Table S2**). The 15-minute group had greater absolute (−0.568 vs. −0.108 ng/mL; P<0.001) and relative declines (−23.7% vs. −8.1%; P = 0.003).

**Table 4:** Change in serum AMH: 5-minute versus 15-minute protocol.

Parameter	5-min (n=124)	15-min (n=84)	Mean difference (95% CI)	P value
Preoperative AMH (ng/mL)	2.11 ± 1.28	1.96 ± 1.33	0.14 (−0.22 to 0.51)	0.440*
Postoperative AMH (ng/mL)	2.00 ± 1.33	1.40 ± 1.02	0.60 (0.28 to 0.93)	<0.001†
Absolute AMH change (ng/mL)	−0.108 ± 0.311	−0.568 ± 0.573	0.46 (0.33 to 0.60)	<0.001†
Relative AMH change (%)	−8.12 ± 19.41	−23.72 ± 51.97	15.60 (5.50 to 25.69)	0.003*
Within-group change, P‡	<0.001	<0.001	—	—

Data are presented as mean ± SD. Absolute AMH change was calculated as postoperative minus preoperative AMH. Relative AMH change was calculated as (postoperative AMH − preoperative AMH)/preoperative AMH × 100%. *Student’s t-test was used when Levene’s test indicated equal variances. †Welch’s t-test was used when Levene’s test indicated unequal variances. ‡Paired t-test comparing postoperative with preoperative AMH within each group. AMH change was not assessed in the expectant group because no intervention-related postoperative assessment was available. AMH, anti-Müllerian hormone; CI, confidence interval.

## Discussion

Laparoscopic cystectomy may reduce the mechanical and inflammatory effects of ovarian endometriomas but can also damage healthy ovarian tissue and compromise ovarian reserve. Ethanol sclerotherapy has therefore been proposed as a less destructive alternative, whereas aspiration alone is limited by a high recurrence rate (23, 24). Since ethanol was initially used to treat renal and hepatic cysts and subsequently applied to ovarian endometriomas, retention times ranging from 1 to 30 minutes have been reported, with 10–15 minutes commonly adopted despite limited comparative evidence (7, 15, 22, 25–27). In the present study, the 5-minute protocol was associated with higher complete cumulative clinical pregnancy and live birth rates than both expectant management and the 15-minute protocol, whereas the 15-minute protocol did not differ significantly from expectant management. These associations were directionally consistent in the doubly robust analyses. However, because treatment allocation and ethanol retention duration were determined clinically rather than randomly assigned, causality cannot be inferred, and residual confounding may still have contributed to the observed differences.Preoperative AMH concentrations were comparable between the two sclerotherapy groups and declined significantly after both procedures, with greater absolute and relative reductions observed after the 15-minute protocol. Previous studies have reported variable changes in AMH after ethanol sclerotherapy, potentially because of differences in procedural approach, ethanol concentration and volume, exposure duration, and the timing of postoperative assessment (16, 28, 29). The association between endometrioma cyst size and serum AMH may also be complex rather than simply inverse. In women without previous surgery for endometriosis, Marcellin et al. reported higher preoperative serum AMH concentrations with increasing endometrioma cyst size, illustrating a potentially paradoxical relationship between cyst size and AMH (30). Although the between-group difference in postoperative AMH change is an observed finding, AMH is a marker of the remaining follicular pool rather than a direct measure of oocyte or embryo quality. In addition, no follow-up AMH measurement was available in the expectant group, and no mediation analysis or direct assessment of ovarian ethanol exposure was performed. Therefore, these findings do not establish a mechanism for the reproductive-outcome differences or prove that 15-minute retention caused greater ovarian injury. Further pharmacokinetic, histological, and longitudinal studies are needed to clarify the clinical relevance and persistence of postoperative AMH changes (26).

Recurrence is another important consideration when comparing exposure protocols. Previous studies comparing sclerotherapy with cystectomy have reported either significantly or numerically higher recurrence after sclerotherapy (31, 32), although shorter ethanol exposure has not consistently been associated with a higher risk of recurrence (33, 34). Recurrence may also be influenced by postoperative medical therapy, subsequent pregnancy, cyst characteristics, and the completeness of ethanol aspiration and cavity irrigation (35, 36). Because recurrence was not assessed systematically over a standardized long-term follow-up period in the present cohort, we could not determine whether the observed between-protocol associations were accompanied by a difference in treatment durability. Future prospective studies should evaluate recurrence, reproductive outcomes, and ovarian reserve simultaneously to define the optimal balance between treatment efficacy and fertility preservation.

The strengths of this study include the simultaneous comparison of expectant management and two ethanol retention durations, complete cumulative reproductive follow-up based on all fresh and frozen embryo transfers derived from the index oocyte retrieval, the use of IPTW and doubly robust analyses, and the combined assessment of cumulative live birth, embryological outcomes, and AMH.

Several limitations should be acknowledged. First, the retrospective, non-randomized design remains susceptible to selection bias, confounding by indication, temporal confounding, and residual unmeasured confounding despite IPTW adjustment, expanded baseline-covariate, calendar-year, and male-factor sensitivity analyses, and doubly robust models. The database did not capture the specific treatment-choice factor or the content and timing of individual clinician-patient discussions. Second, the weighted effective sample size of the 15-minute group was limited, reducing precision; consequently, nonsignificant comparisons with expectant management should not be interpreted as evidence of equivalence. Third, we reconstructed pathway-specific fresh/frozen transfer outcomes and report them descriptively. Because women could contribute repeated transfer cycles and to both pathways, these data do not provide independent pathway-specific observations, were not used for causal pathway comparisons, and do not replace the prespecified patient-level cumulative outcomes. Fourth, because oocyte maturity was not assessed before conventional IVF insemination but was identified before ICSI injection, a common mature-oocyte or injected-oocyte denominator was not applicable across both fertilization methods. The 2PN endpoint was therefore expressed as yield per retrieved oocyte. Fifth, embryological outcomes from the 2PN stage onward were adjusted for fertilization method, whereas the outcome of no transferable embryo was analyzed using IPTW alone. Embryo quality was evaluated morphologically without genetic or functional assessment. Sixth, AMH changes were assessed only in the two sclerotherapy groups, without a corresponding follow-up measurement in the expectant group or long-term serial measurements. Finally, recurrence, detailed cyst-wall histopathology, and direct histological evidence of ethanol-related ovarian injury were not systematically evaluated. Prospective multicenter studies with randomized exposure duration, standardized follow-up, transfer-level outcome linkage, and longitudinal reproductive and ovarian-reserve assessment are required.

## Conclusion

In this retrospective cohort, the 5-minute ethanol-retention protocol was associated with higher cumulative clinical pregnancy and live birth rates than expectant management and the 15-minute protocol. Both sclerotherapy protocols were associated with fewer cycles yielding no transferable embryo than expectant management, whereas 15-minute retention was associated with a greater AMH decline. These findings are hypothesis-generating and require confirmation in adequately powered prospective randomized studies.

## Data Availability

The datasets analyzed during the current study are available from the corresponding author on reasonable request.

## References

[1] Taylor HS Kotlyar AM Flores VA . Endometriosis is a chronic systemic disease: clinical challenges and novel innovations. Lancet. (2021) 397:839–52. doi: 10.1016/s0140-6736(21)00389-5 33640070

[2] Muzii L Di Tucci C Di Feliciantonio M Galati G Verrelli L Donato VD . Management of endometriomas. Semin Reprod Med. (2017) 35:25–30. doi: 10.1055/s-0036-1597126 27926971

[3] Orazov MR Radzinsky VY Ivanov II Khamoshina MB Shustova VB . Oocyte quality in women with infertility associated endometriosis. Gynecol Endocrinol. (2019) 35:24–6. doi: 10.1080/09513590.2019.1632088 31532315

[4] Fox C Morin S Jeong JW Scott RT Jr Lessey BA . Local and systemic factors and implantation: what is the evidence? Fertil Steril. (2016) 105:873–84. doi: 10.1016/j.fertnstert.2016.02.018 26945096 PMC4821679

[5] Younis JS Taylor HS . The impact of ovarian endometrioma and endometriotic cystectomy on anti-Müllerian hormone, and antral follicle count: a contemporary critical appraisal of systematic reviews. Front Endocrinol (Lausanne). (2024) 15:1397279. doi: 10.3389/fendo.2024.1397279 38800489 PMC11116636

[6] Younis JS Shapso N Ben-Sira Y Nelson SM Izhaki I . Endometrioma surgery-a systematic review and meta-analysis of the effect on antral follicle count and anti-Müllerian hormone. Am J Obstet Gynecol. (2022) 226:33–51.e37. doi: 10.1016/j.ajog.2021.06.102 34265271

[7] Akamatsu N Hirai T Masaoka H Sekiba K Fujita T . Ultrasonically guided puncture of endometrial cysts--aspiration of contents and infusion of ethanol. Nihon Sanka Fujinka Gakkai Zasshi. (1988) 40:187–91. 3283269

[8] Zeng CH Cao CW Shin JH Kim GH Kim SH Lee SR . Safety and clinical outcomes of two-session catheter-directed sclerotherapy using ethanol for endometrioma. Cardiovasc Intervent Radiol. (2024) 47:901–9. doi: 10.1007/s00270-024-03700-5 38528174

[9] Lee JK Han K Choi E Baek J Kim HR Kim MD . Effect of catheter-directed ethanol sclerotherapy on ovarian reserve in patients with recurrent endometrioma: comparative analysis with primary endometriosis. Eur Radiol. (2024) 34:3298–308. doi: 10.1007/s00330-023-10320-z 37848771

[10] Mohtashami S Jabarpour M Aleyasin A Aghahosseini M Najafian A . Efficacy of ethanol sclerotherapy versus laparoscopic excision in the treatment of ovarian endometrioma. J Obstet Gynaecol India. (2024) 74:60–6. doi: 10.1007/s13224-023-01840-1 38434126 PMC10902231

[11] Muzii L Marana R Caruana P Catalano GF Mancuso S . Laparoscopic findings after transvaginal ultrasound-guided aspiration of ovarian endometriomas. Hum Reprod. (1995) 10:2902–3. doi: 10.1093/oxfordjournals.humrep.a135815 8747040

[12] De Cicco Nardone A Carfagna P De Cicco Nardone C Scambia G Marana R De Cicco Nardone F . Laparoscopic ethanol sclerotherapy for ovarian endometriomas: preliminary results. J Minim Invasive Gynecol. (2020) 27:1331–6. doi: 10.1016/j.jmig.2019.09.792 32380241

[13] Şükür YE Aslan B Varlı B Özcan P Daniilidis A Kalaitzopoulos DR . Ethanol sclerotherapy for endometriomas in infertile women: a narrative review. J Clin Med. (2024) 13(24):7548. doi: 10.3390/jcm13247548 PMC11727901 39768471

[14] Becker CM Bokor A Heikinheimo O Horne A Jansen F Kiesel L . ESHRE guideline: endometriosis. Hum Reprod Open. (2022) 2022:hoac009. doi: 10.1093/hropen/hoac009 35350465 PMC8951218

[15] Ronsini C Iavarone I Braca E Vastarella MG De Franciscis P Torella M . The efficiency of sclerotherapy for the management of endometrioma: a systematic review and meta-analysis of clinical and fertility outcomes. Med (Kaunas). (2023) 59(9):1643. doi: 10.3390/medicina59091643 37763762 PMC10535205

[16] Huang L Chang MY Shiau CS Hsieh TT . Changes in anti-müllerian hormone after ultrasound guided aspiration and ethanol sclerotic therapy of ovarian cyst. Taiwan J Obstet Gynecol. (2021) 60:509–12. doi: 10.1016/j.tjog.2021.03.021 33966737

[17] Koike T Minakami H Motoyama M Ogawa S Fujiwara H Sato I . Reproductive performance after ultrasound-guided transvaginal ethanol sclerotherapy for ovarian endometriotic cysts. Eur J Obstet Gynecol Reprod Biol. (2002) 105:39. doi: 10.1016/s0301-2115(02)00144-6 12270563

[18] Meng B Xu M . Pelvic artificial isolation with ultrasound-guided fluid: a new technique in ovarian endometriotic cyst transabdominal sclerotherapy. Med Sci Monit. (2022) 28:e937855. doi: 10.12659/msm.937855 36303417 PMC9624109

[19] Kitajima M Dolmans MM Donnez O Masuzaki H Soares M Donnez J . Enhanced follicular recruitment and atresia in cortex derived from ovaries with endometriomas. Fertil Steril. (2014) 101:1031–7. doi: 10.1016/j.fertnstert.2013.12.049 24502890

[20] Dunselman GA Vermeulen N Becker C Calhaz-Jorge C D'Hooghe T De Bie B . ESHRE guideline: management of women with endometriosis. Hum Reprod. (2014) 29:400–12. doi: 10.1093/humrep/det457 24435778

[21] Harmsen MJ Van den Bosch T de Leeuw RA Dueholm M Exacoustos C Valentin L . Consensus on revised definitions of Morphological Uterus Sonographic Assessment (MUSA) features of adenomyosis: results of modified Delphi procedure. Ultrasound Obstet Gynecol. (2022) 60:118–31. doi: 10.1002/uog.24786 34587658 PMC9328356

[22] Crestani A Merlot B Dennis T Roman H . Laparoscopic sclerotherapy for an endometrioma in 10 steps. Fertil Steril. (2022) 117:1102–3. doi: 10.1016/j.fertnstert.2022.01.036 35351292

[23] Wang LL Dong XQ Shao XH Wang SM . Ultrasound-guided interventional therapy for recurrent ovarian chocolate cysts. Ultrasound Med Biol. (2011) 37:1596–602. doi: 10.1016/j.ultrasmedbio.2011.07.004 21856071

[24] Marana R Caruana P Muzii L Catalano GF Mancuso S . Operative laparoscopy for ovarian cysts. excision vs. aspiration. J Reprod Med. (1996) 41:435–8. doi: 10.1097/00006254-199610000-00012 8799921

[25] Bean WJ . Renal cysts: treatment with alcohol. Radiology. (1981) 138:329–31. doi: 10.1148/radiology.138.2.7455112 7455112

[26] Bean WJ Rodan BA . Hepatic cysts: treatment with alcohol. AJR Am J Roentgenol. (1985) 144:237–41. doi: 10.2214/ajr.144.2.237 3880981

[27] Miquel L Preaubert L Gnisci A Netter A Courbiere B Agostini A . Transvaginal ethanol sclerotherapy for an endometrioma in 10 steps. Fertil Steril. (2021) 115:259–60. doi: 10.1016/j.fertnstert.2020.08.1422 33039127

[28] Crestani A Merlot B Dennis T Chanavaz-Lacheray I Roman H . Impact of laparoscopic sclerotherapy for ovarian endometriomas on ovarian reserve. J Minim Invasive Gynecol. (2023) 30:32–8. doi: 10.1016/j.jmig.2022.10.001 36228863

[29] Koo JH Lee I Han K Seo SK Kim MD Lee JK . Comparison of the therapeutic efficacy and ovarian reserve between catheter-directed sclerotherapy and surgical excision for ovarian endometrioma. Eur Radiol. (2021) 31:543–8. doi: 10.1007/s00330-020-07111-1 32770376

[30] Marcellin L Santulli P Bourdon M Comte C Maignien C Just PA . Serum antimüllerian hormone concentration increases with ovarian endometrioma size. Fertil Steril. (2019) 111:944–952.e941. doi: 10.1016/j.fertnstert.2019.01.013 30878253

[31] Alborzi S Askary E Keramati P Moradi Alamdarloo S Poordast T Ashraf MA . Assisted reproductive technique outcomes in patients with endometrioma undergoing sclerotherapy vs laparoscopic cystectomy: prospective cross-sectional study. Reprod Med Biol. (2021) 20:313–20. doi: 10.1002/rmb2.12386 34262399 PMC8254172

[32] Noma J Yoshida N . Efficacy of ethanol sclerotherapy for ovarian endometriomas. Int J Gynaecol Obstet. (2001) 72:35–9. doi: 10.1016/s0020-7292(00)00307-6 11146075

[33] Ikuta A Tanaka Y Mizokami T Tsutsumi A Sato M Tanaka M . Management of transvaginal ultrasound-guided absolute ethanol sclerotherapy for ovarian endometriotic cysts. J Med Ultrason (2001). (2006) 33:99–103. doi: 10.1007/s10396-005-0079-2 27277728

[34] André GM Vilarino FL Christofolini DM Bianco B Barbosa CP . Aspiration and ethanol sclerotherapy to treat recurrent ovarian endometriomas prior to *in vitro* fertilization – a pilot study. Einstein (Sao Paulo). (2011) 9:494–8. doi: 10.1590/S1679-45082011AO2081 26761252

[35] Koshiba A Mori T Okimura H Akiyama K Kataoka H Takaoka O . Dienogest therapy during the early stages of recurrence of endometrioma might be an alternative therapeutic option to avoid repeat surgeries. J Obstet Gynaecol Res. (2018) 44:1970–6. doi: 10.1111/jog.13725 29992672

[36] Yamanaka A Hada T Matsumoto T Kanno K Shirane A Yanai S . Effect of dienogest on pain and ovarian endometrioma occurrence after laparoscopic resection of uterosacral ligaments with deep infiltrating endometriosis. Eur J Obstet Gynecol Reprod Biol. (2017) 216:51–5. doi: 10.1016/j.ejogrb.2017.07.014 28728071

