# Supplementary material for: Reproductive outcomes after expectant management and 5- versus 15-minute laparoscopic ethanol sclerotherapy for ovarian endometriomas: a retrospective cohort study
Source: Front Endocrinol (Lausanne). 2026 Sep 28;17:1959548. doi: 10.3389/fendo.2026.1959548 (PMC13654783; doi:10.3389/fendo.2026.1959548)
Supplement: Supplementary file 1 [file Table1.docx]

**Supplementary Table 1. Stimulation, fertilization, and embryo-transfer characteristics and exploratory transfer-pathway reproductive outcomes**

**Panel A. Stimulation parameters, fertilization methods, and embryo-transfer characteristics (333 women with complete cumulative outcomes)**

| **Characteristic** | **Expectant (n=125)** | **5-min (n=124)** | **15-min (n=84)** | **P value** |
| --- | --- | --- | --- | --- |
| **Stimulation parameters** |  |  |  |  |
| Basal FSH (IU/L) | 8.14 ± 2.73 | 7.76 ± 2.82 | 7.83 ± 2.08 | 0.311 |
| Total gonadotropin dose (IU) | 2293.60 ± 867.84 | 2448.19 ± 887.65 | 2682.24 ± 835.08 | 0.007 |
| Stimulation duration (days) | 9.26 ± 2.30 | 10.17 ± 2.39 | 10.94 ± 2.43 | <0.001 |
| **Fertilization method, n (%)** |  |  |  | 0.347 |
| IVF | 87 (69.6) | 92 (74.2) | 66 (78.6) |  |
| ICSI | 38 (30.4) | 32 (25.8) | 18 (21.4) |  |
| **Patient-level ET cycles** |  |  |  |  |
| Total ET cycles per woman | 0.97 ± 1.09 | 1.23 ± 0.71 | 1.11 ± 0.74 | 0.082 |
| Fresh ET cycles per woman | 0.24 ± 0.43 | 0.60 ± 0.49 | 0.52 ± 0.50 | <0.001 |
| Frozen-thawed ET cycles per woman | 0.73 ± 1.06 | 0.63 ± 0.84 | 0.58 ± 0.79 | 0.497 |
| Cleavage-stage ET cycles per woman | 0.50 ± 0.59 | 0.70 ± 0.51 | 0.60 ± 0.49 | 0.011 |
| Blastocyst ET cycles per woman | 0.47 ± 0.93 | 0.52 ± 0.73 | 0.51 ± 0.80 | 0.875 |
| Single-embryo ET cycles per woman | 0.45 ± 0.56 | 0.60 ± 0.73 | 0.63 ± 0.58 | 0.064 |
| Double-embryo ET cycles per woman | 0.52 ± 0.90 | 0.62 ± 0.56 | 0.48 ± 0.59 | 0.315 |
| **Aggregate ET cycle counts, n** |  |  |  |  |
| Total ET cycles | 121 | 152 | 93 |  |
| Fresh ET cycles | 30 | 74 | 44 |  |
| Frozen-thawed ET cycles | 91 | 78 | 49 |  |
| Cleavage-stage ET cycles | 62 | 87 | 50 |  |
| Blastocyst ET cycles | 59 | 65 | 43 |  |
| Single-embryo ET cycles | 56 | 75 | 53 |  |
| Double-embryo ET cycles | 65 | 77 | 40 |  |
| Women with >=1 ET, n (%) | 78 (62.4) | 113 (91.1) | 71 (84.5) |  |

**Panel B. Exploratory transfer-level reproductive outcomes (same 333 women with complete cumulative outcomes)**

| **Characteristic** | **Expectant (n=125)** | **5-min (n=124)** | **15-min (n=84)** |
| --- | --- | --- | --- |
| Women with complete cumulative outcomes, n | 125 | 124 | 84 |
| **Fresh transfer pathway** |  |  |  |
| Women contributing, n | 30 | 74 | 44 |
| Transfer cycles, n | 30 | 74 | 44 |
| Clinical pregnancy, n/N (%) | 11/30 (36.7%) | 55/74 (74.3%) | 25/44 (56.8%) |
| Live birth, n/N (%) | 6/30 (20.0%) | 50/74 (67.6%) | 19/44 (43.2%) |
| **Frozen-thawed transfer pathway** |  |  |  |
| Women contributing, n | 63 | 54 | 37 |
| Transfer cycles, n | 91 | 78 | 49 |
| Clinical pregnancy, n/N (%) | 48/91 (52.7%) | 54/78 (69.2%) | 27/49 (55.1%) |
| Live birth, n/N (%) | 41/91 (45.1%) | 45/78 (57.7%) | 24/49 (49.0%) |

Panel A and Panel B include the same 333 women with complete cumulative outcomes (125 expectant, 124 5-minute, and 84 15-minute). Panel A data are mean ± SD or n (%). P values in Panel A were calculated from unweighted data and are descriptive. Continuous variables were compared using one-way ANOVA or Welch's ANOVA according to Levene's test; categorical variables were compared using chi-square tests. Patient-level ET summaries are descriptive; aggregate ET cycle counts were not treated as independent observations. The unit of analysis in Panel B is the transfer cycle. Clinical pregnancy includes transfers ending in miscarriage or live birth. Women could contribute more than one transfer cycle and, where applicable, to both fresh and frozen-thawed pathway summaries. Panel B rates are descriptive; therefore, no P values or pathway-specific regression models are reported. FSH, follicle-stimulating hormone; ET, embryo transfer; IVF, in vitro fertilization; ICSI, intracytoplasmic sperm injection; SD, standard deviation.

**Supplementary Table 2. Surgical-stage and postoperative AMH assessment characteristics**

| **Characteristic** | **5-min (n=124)** | **15-min (n=84)** |
| --- | --- | --- |
| **rASRM stage, n (%)** |  |  |
| Stage II | 0 (0.0) | 1 (1.2) |
| Stage III | 48 (38.7) | 27 (32.1) |
| Stage IV | 76 (61.3) | 56 (66.7) |
| **Interval from surgery to AMH assessment (days)** |  |  |
| Median (IQR) | 59.0 (52.0–66.25) | 54.5 (49.0–64.0) |
| Mean ± SD | 59.77 ± 9.35 | 56.93 ± 9.31 |
| Range | 42–82 | 42–78 |

Data are presented as n (%) or summary statistics. rASRM staging and postoperative AMH timing were reported only for the surgical groups. AMH, anti-Müllerian hormone; IQR, interquartile range; rASRM, revised American Society for Reproductive Medicine classification; SD, standard deviation.

**Supplementary Table 3. Sensitivity analyses of cumulative reproductive outcomes**

**Panel A. IPTW-weighted rates and pairwise comparisons**

| **Sensitivity model** | **n** | **Outcome** | **Expectant (%)** | **5-min (%)** | **15-min (%)** | **Overall P*** | **Comparison** | **OR (95% CI)** | **P value*** |
| --- | --- | --- | --- | --- | --- | --- | --- | --- | --- |
| Expanded baseline (FSH + adenomyosis) | 333 | CPR | 48.2 | 78.5 | 57.9 | <0.001 | 5-min vs. Expectant | 3.91 (1.89–8.10) | <0.001 |
| Expanded baseline (FSH + adenomyosis) | 333 | CPR |  |  |  |  | 15-min vs. Expectant | 1.48 (0.68–3.22) | 0.474 |
| Expanded baseline (FSH + adenomyosis) | 333 | CPR |  |  |  |  | 5-min vs. 15-min | 2.65 (1.15–6.11) | 0.017 |
| Expanded baseline (FSH + adenomyosis) | 333 | CLBR | 42.8 | 73.2 | 50.8 | <0.001 | 5-min vs. Expectant | 3.65 (1.81–7.35) | <0.001 |
| Expanded baseline (FSH + adenomyosis) | 333 | CLBR |  |  |  |  | 15-min vs. Expectant | 1.38 (0.63–3.01) | 0.598 |
| Expanded baseline (FSH + adenomyosis) | 333 | CLBR |  |  |  |  | 5-min vs. 15-min | 2.64 (1.19–5.86) | 0.012 |
| Calendar year | 333 | CPR | 49.2 | 78.5 | 59.5 | <0.001 | 5-min vs. Expectant | 3.77 (1.79–7.95) | <0.001 |
| Calendar year | 333 | CPR |  |  |  |  | 15-min vs. Expectant | 1.52 (0.64–3.57) | 0.490 |
| Calendar year | 333 | CPR |  |  |  |  | 5-min vs. 15-min | 2.49 (0.99–6.24) | 0.053 |
| Calendar year | 333 | CLBR | 43.7 | 73.0 | 50.8 | <0.001 | 5-min vs. Expectant | 3.49 (1.70–7.14) | <0.001 |
| Calendar year | 333 | CLBR |  |  |  |  | 15-min vs. Expectant | 1.33 (0.57–3.11) | 0.712 |
| Calendar year | 333 | CLBR |  |  |  |  | 5-min vs. 15-min | 2.62 (1.09–6.30) | 0.027 |
| Male factor | 333 | CPR | 48.8 | 78.2 | 58.3 | <0.001 | 5-min vs. Expectant | 3.77 (1.82–7.80) | <0.001 |
| Male factor | 333 | CPR |  |  |  |  | 15-min vs. Expectant | 1.47 (0.67–3.21) | 0.480 |
| Male factor | 333 | CPR |  |  |  |  | 5-min vs. 15-min | 2.57 (1.11–5.92) | 0.022 |
| Male factor | 333 | CLBR | 43.0 | 73.0 | 51.0 | <0.001 | 5-min vs. Expectant | 3.58 (1.78–7.21) | <0.001 |
| Male factor | 333 | CLBR |  |  |  |  | 15-min vs. Expectant | 1.38 (0.63–3.00) | 0.598 |
| Male factor | 333 | CLBR |  |  |  |  | 5-min vs. 15-min | 2.60 (1.17–5.77) | 0.014 |
| Conservative lower-bound | 357 | CPR | 50.2 | 78.6 | 59.2 | <0.001 | 5-min vs. Expectant | 3.63 (1.80–7.32) | <0.001 |
| Conservative lower-bound | 357 | CPR |  |  |  |  | 15-min vs. Expectant | 1.44 (0.68–3.03) | 0.493 |
| Conservative lower-bound | 357 | CPR |  |  |  |  | 5-min vs. 15-min | 2.53 (1.12–5.70) | 0.021 |
| Conservative lower-bound | 357 | CLBR | 37.2 | 68.3 | 49.2 | <0.001 | 5-min vs. Expectant | 3.62 (1.88–6.98) | <0.001 |
| Conservative lower-bound | 357 | CLBR |  |  |  |  | 15-min vs. Expectant | 1.63 (0.77–3.46) | 0.278 |
| Conservative lower-bound | 357 | CLBR |  |  |  |  | 5-min vs. 15-min | 2.22 (1.05–4.72) | 0.035 |

All models used multinomial propensity-score weighting with stabilized weights truncated at the 1st and 99th percentiles. Panel A follows Main Table 3, Panel A: rates are IPTW-weighted estimates, overall P values are design-based Wald tests, and pairwise ORs are shown in the direction listed; an OR >1 indicates higher odds in the first-listed group. The expanded baseline-covariate, calendar-year, and male-factor models included 333 women with complete cumulative outcomes. The conservative lower-bound model included all 357 clinically eligible women. All outcomes from completed transfer cycles were observed. For women who had not achieved a live birth but retained unused cryopreserved embryos at database closure, the potential contribution of future transfers to cumulative live birth was conservatively assumed to be zero. This represents a worst-case lower-bound assumption and does not eliminate informative follow-up.

**Panel B. Doubly robust models (reference = Expectant)**

| **Sensitivity model** | **n** | **Outcome** | **Variable** | **β (SE)** | **P value*** |
| --- | --- | --- | --- | --- | --- |
| Expanded baseline (FSH + adenomyosis) | 333 | CPR | 5-min group | 1.571 (0.339) | P<0.001 |
| Expanded baseline (FSH + adenomyosis) | 333 | CPR | 15-min group | 0.412 (0.358) | P=0.250 |
| Expanded baseline (FSH + adenomyosis) | 333 | CLBR | 5-min group | 1.540 (0.329) | P<0.001 |
| Expanded baseline (FSH + adenomyosis) | 333 | CLBR | 15-min group | 0.371 (0.363) | P=0.307 |
| Calendar year | 333 | CPR | 5-min group | 1.544 (0.350) | P<0.001 |
| Calendar year | 333 | CPR | 15-min group | 0.501 (0.392) | P=0.202 |
| Calendar year | 333 | CLBR | 5-min group | 1.504 (0.344) | P<0.001 |
| Calendar year | 333 | CLBR | 15-min group | 0.384 (0.399) | P=0.337 |
| Male factor | 333 | CPR | 5-min group | 1.543 (0.340) | P<0.001 |
| Male factor | 333 | CPR | 15-min group | 0.398 (0.356) | P=0.264 |
| Male factor | 333 | CLBR | 5-min group | 1.529 (0.331) | P<0.001 |
| Male factor | 333 | CLBR | 15-min group | 0.361 (0.360) | P=0.317 |
| Conservative lower-bound | 357 | CPR | 5-min group | 1.463 (0.327) | P<0.001 |
| Conservative lower-bound | 357 | CPR | 15-min group | 0.366 (0.344) | P=0.288 |
| Conservative lower-bound | 357 | CLBR | 5-min group | 1.479 (0.305) | P<0.001 |
| Conservative lower-bound | 357 | CLBR | 15-min group | 0.545 (0.339) | P=0.109 |

Panel B follows Main Table 3, Panel B. Doubly robust models combined IPTW with outcome-model adjustment for covariates in the corresponding sensitivity model. β values are on the log-odds scale; the 5-min and 15-min group coefficients use expectant management as the reference group. Male factor was included as an additional binary sensitivity covariate and was not added to the prespecified primary model.

**Supplementary Table 4. Weighting and propensity-score diagnostics**

**Panel A. Covariate balance, effective sample size, and stabilized-weight summaries**

| **Model** | **n** | **Max absolute post-IPTW SMD** | **All <0.10** | **ESS Expectant** | **ESS 5-min** | **ESS 15-min** | **Truncated stabilized-weight range** | **Median weight** | **99th percentile weight** |
| --- | --- | --- | --- | --- | --- | --- | --- | --- | --- |
| Expanded baseline (FSH + adenomyosis) | 333 | 0.0979 | Yes | 99.7 | 117.7 | 58.8 | 0.414–3.107 | 0.896 | 3.106 |
| Calendar year | 333 | 0.0997 | Yes | 100.0 | 111.1 | 45.1 | 0.311–4.632 | 0.834 | 4.280 |
| Male factor | 333 | 0.0998 | Yes | 100.8 | 117.5 | 58.6 | 0.405–3.188 | 0.897 | 3.140 |
| Conservative lower-bound | 357 | 0.0874 | Yes | 112.9 | 125.1 | 62.8 | 0.398–2.944 | 0.898 | 2.881 |

The maximum absolute post-IPTW SMD is calculated across included covariates and all three pairwise treatment-group comparisons; values <0.10 were considered adequate. ESS, effective sample size. Weight ranges are after 1st/99th percentile truncation.

**Panel B. Observed-treatment propensity-score overlap**

| **Model** | **Observed treatment group** | **n** | **Observed-treatment propensity-score range** | **25th percentile** | **Median** | **75th percentile** | **Excluded for lack of overlap, n** |
| --- | --- | --- | --- | --- | --- | --- | --- |
| Expanded baseline (FSH + adenomyosis) | Expectant | 125 | 0.121–0.732 | 0.331 | 0.439 | 0.579 | 0 |
| Expanded baseline (FSH + adenomyosis) | 5-min | 124 | 0.231–0.642 | 0.331 | 0.394 | 0.456 | 0 |
| Expanded baseline (FSH + adenomyosis) | 15-min | 84 | 0.061–0.774 | 0.195 | 0.300 | 0.492 | 0 |
| Calendar year | Expectant | 125 | 0.106–0.821 | 0.330 | 0.462 | 0.584 | 0 |
| Calendar year | 5-min | 124 | 0.151–0.665 | 0.330 | 0.421 | 0.512 | 0 |
| Calendar year | 15-min | 84 | 0.027–0.844 | 0.196 | 0.388 | 0.527 | 0 |
| Male factor | Expectant | 125 | 0.124–0.738 | 0.333 | 0.431 | 0.567 | 0 |
| Male factor | 5-min | 124 | 0.229–0.636 | 0.332 | 0.393 | 0.464 | 0 |
| Male factor | 15-min | 84 | 0.048–0.771 | 0.191 | 0.332 | 0.491 | 0 |
| Conservative lower-bound | Expectant | 136 | 0.135–0.727 | 0.327 | 0.451 | 0.559 | 0 |
| Conservative lower-bound | 5-min | 133 | 0.204–0.667 | 0.323 | 0.386 | 0.465 | 0 |
| Conservative lower-bound | 15-min | 88 | 0.048–0.795 | 0.183 | 0.344 | 0.476 | 0 |

Propensity-score ranges summarize the predicted probability of the treatment actually received within each observed treatment group. No observations were excluded for lack of overlap in these analyses.

**Supplementary Table 5. Annual distribution of management strategies in the clinically eligible cohort**

| **Calendar year** | **Expectant, n** | **5-min, n** | **15-min, n** | **Total, n** |
| --- | --- | --- | --- | --- |
| 2019 | 6 | 14 | 8 | 28 |
| 2020 | 23 | 15 | 12 | 50 |
| 2021 | 22 | 15 | 32 | 69 |
| 2022 | 23 | 31 | 10 | 64 |
| 2023 | 31 | 20 | 11 | 62 |
| 2024 | 31 | 38 | 15 | 84 |

Counts are shown for all 357 clinically eligible women. The protocols were used contemporaneously during 2019–2024; calendar year was evaluated as a categorical covariate in a separate sensitivity analysis.

**Supplementary Table 6. Follow-up duration and completeness in the clinically eligible cohort**

| **Group** | **n** | **Median follow-up, days (IQR)** | **Complete follow-up, n (%)** | **Incomplete follow-up, n (%)** |
| --- | --- | --- | --- | --- |
| Expectant | 136 | 123.0 (12.0–346.0) | 125 (91.9%) | 11 (8.1%) |
| 5-min | 133 | 332.0 (302.0–363.0) | 124 (93.2%) | 9 (6.8%) |
| 15-min | 88 | 303.0 (94.8–342.2) | 84 (95.5%) | 4 (4.5%) |

Follow-up duration was calculated from index oocyte retrieval to the earliest of first live birth, use of all embryos derived from the index retrieval, or database closure. Complete follow-up indicates that the cumulative outcome had matured; incomplete follow-up indicates that unused cryopreserved embryos remained at database closure.
